# Effects of PUMILIO1 and PUMILIO2 knockdown on cardiomyogenic differentiation of human embryonic stem cells culture

**DOI:** 10.1371/journal.pone.0222373

**Published:** 2020-05-21

**Authors:** Isabelle Leticia Zaboroski Silva, Anny Waloski Robert, Guillermo Cabrera Cabo, Lucia Spangenberg, Marco Augusto Stimamiglio, Bruno Dallagiovanna, Daniela Fiori Gradia, Patrícia Shigunov

**Affiliations:** 1 Laboratory of Basic Biology of Stem Cells (LABCET), Instituto Carlos Chagas—FIOCRUZ-PR, Curitiba, Paraná, Brazil; 2 Bioinformatics Unit, Instituto Pasteur de Montevideo, Montevideo, Uruguay; 3 Department of Genetics, Federal University of Parana (UFPR), Curitiba, Paraná, Brazil; Nanjing Medical University, CHINA

## Abstract

Posttranscriptional regulation plays a fundamental role in the biology of embryonic stem cells (ESCs). Many studies have demonstrated that multiple mRNAs are coregulated by one or more RNA-binding proteins (RBPs) that orchestrate mRNA expression. A family of RBPs, which is known as the Pumilio-FBF (PUF) family, is highly conserved among different species and has been associated with the undifferentiated and differentiated states of different cell lines. In humans, two homologs of the PUF family have been found: Pumilio 1 (PUM1) and Pumilio 2 (PUM2). To understand the role of these proteins in human ESCs (hESCs), we first assessed the influence of the silencing of *PUM1* and *PUM2* on pluripotency genes and found that the knockdown of Pumilio genes significantly decreased the *OCT4* and *NANOG* mRNA levels and reduced the amount of nuclear OCT4, which suggests that Pumilio proteins play a role in the maintenance of pluripotency in hESCs. Furthermore, we observed that *PUM1*-and-*PUM2*-silenced hESCs exhibited improved efficiency of *in vitro* cardiomyogenic differentiation. Through an *in silico* analysis, we identified mRNA targets of PUM1 and PUM2 that are expressed at the early stages of cardiomyogenesis, and further investigation will determine whether these target mRNAs are active and involved in the progression of cardiomyogenesis. Our findings contribute to the understanding of the role of Pumilio proteins in hESC maintenance and differentiation.

## Introduction

Human embryonic stem cells (hESCs) are pluripotent cells derived from the inner cell mass of the blastocyst that have the potential to differentiate into cells belonging to each of the three germ layers [1–3]⁠. In an undifferentiated state, hESCs are characterized by the expression of stemness factors such as OCT4 (POU5F1), SOX2 and NANOG [4]. These three transcription factors, which are positively regulated, are responsible for the maintenance of pluripotency and contribute to the repression of lineage-specific genes [reviewed by 5]. The stimulation of hESCs to undergo the differentiation process decreases the expression of genes associated with pluripotency and initiates the expression of genes associated with the germ layer [6].

A complex network of gene expression underlies the molecular signaling that gives rise to different organs and tissues, including the heart. Cardiomyogenic differentiation is a highly regulated process that depends on the finely tuned regulation of gene expression [7]. The *in vitro* cardiomyogenic differentiation of hESCs can be used as a model for studying cardiac development and electrophysiology as well as for drug screening and the development of potential cellular therapies [reviewed by 8].

RNA-binding proteins (RBPs) are proteins that contain RNA-binding domains and form ribonucleoprotein complexes in association with RNAs (RNPs). These proteins play a critical role in the posttranscriptional regulation of gene expression. The dynamics and functions of these complexes depend on their composition, targets and cofactors [9]. The Pumilio-FBF (PUF) family of RBPs is highly conserved among species and is found in plants, insects, nematodes and mammals [10–15]. The RNA-interaction domain of Pumilio proteins is highly conserved [16] and comprises eight repeats, each of which has the ability to bind a single nucleotide of a specific recognition motif in the 3′ untranslated region (UTR) of a target mRNA [17]. In humans, there are two homologs of the PUF family, PUMILIO 1 (PUM1) and PUMILIO 2 (PUM2), which exhibit 91% identity in their RNA-binding domains [15].

The expression of PUM1 and PUM2 has been detected in hESCs and several human fetal and adult tissues, including the ovaries and testes [11,12]. Furthermore, in mammals, the disruption of PUM1 causes defective germline phenotypes [18,19]. In rodents, Pum1 facilitates the exit of cells from an undifferentiated state to a differentiated form by accelerating the degradation of some important factors that maintain pluripotency, such as Tfcp2l1, Sox2, Tbx3, and Esrrb [20]. In addition, many of the mRNAs associated with PUM1 belong to a relatively small number of functional groups, which suggests the existence of an RNA regulon model [21] in which PUM1 inhibits translation and promotes the degradation of its target mRNAs [22]. Pumilio proteins form multiprotein complexes with other regulatory proteins, such as DAZ-like (DAZL) [11], boule (BOL) [23], staufen (STAU) [24] and nanos (NOS) [25]. These complexes are also involved in the maintenance of ESCs and in the regulation of the onset of meiosis in various organisms, including humans [11,26]. PUM2 and NOS interact in a conserved mechanism to participate in the differentiation and maintenance of germ cells [25]. Thus, the molecular mechanisms underlying the functions of Pumilio proteins and their targets might determine whether cells undergo differentiation or maintain a stemness phenotype.

Here, we investigated the roles of PUM1 and PUM2 proteins in hESCs in the maintenance of pluripotency and during cardiomyogenic differentiation. We found that the silencing of *PUM1* and *PUM2* in hESCs reduced the expression of pluripotency genes and positively influenced cardiomyogenesis. Moreover, based on an *in silico* analysis, we selected targets of PUM1 and PUM2 from previously published polysome-bound mRNA data [27] and identified biological processes and gene networks related to Pumilio proteins that occur during the early stages of *in vitro* cardiac differentiation.

## Methods

### Cell culture and cardiac differentiation

The *NKX2-5*^*eGFP/w*^ HES3 hESC line [28] was generously provided by Monash University (Victoria, Australia). The cell cultures were maintained on irradiated mouse embryonic fibroblasts (iMEFs) in hESC medium consisting of Dulbecco’s modified Eagle’s medium (DMEM)/F12 supplemented with 20% KnockOut serum replacement, 1% nonessential amino acids, 1% L-glutamine, 1% penicillin/streptomycin, 0.1 mM β-mercaptoethanol and 10 ng/ml human βFGF. The cells were passaged every 3 to 4 days by enzymatic dissociation using 0.25% trypsin/EDTA. Cardiomyogenic differentiation assays were conducted using an embryoid body (EB) protocol adapted from previously described methods [27,29] or a previously reported monolayer protocol [30].

For EB cardiac differentiation, 7x10^5^ cells/well were plated in six-well dishes containing growth factor-reduced Matrigel^®^ Matrix (Corning). Then, hESCs were dissociated and cultured on low-attachment plates supplemented with StemPro-34 medium (composed of StemPro™-34 SFM (Gibco™) supplemented with transferrin, ascorbic acid, penicillin/streptomycin and monothioglycerol) containing BMP4 (0.5 ng/ml) for 24 h to form EBs (Day 0, D0). On day 1 (D1), the EBs were incubated with StemPro-34 supplemented with βFGF (5 ng/ml), activin A (6 ng/ml) and BMP4 (10 ng/ml) to induce mesoderm differentiation. On day 4 (D4), the EBs were incubated with medium supplemented with XAV939 (10 μM/ml) and VEGF (10 ng/ml) to induce differentiation of the cells into cardiac progenitors. On days 8 and 11, the medium was changed to StemPro-34 containing only VEGF (10 ng/ml). On day 9, it was possible to verify the percentage of cells that were committed to cardiac differentiation based on eGFP expression (relative to *NKX2*.*5* expression). On day 15 (D15), we evaluated the efficiency of the protocol through cardiac troponin T (cTnT) staining. During the differentiation period, the EBs were maintained in a humidified incubator under hypoxic conditions (5% O_2_, 5% CO_2_, 37°C).

For monolayer cardiac differentiation, hESCs were dissociated from iMEF cultures, and 1.5x10^5^ cells/well were plated into Matrigel^®^ hESC-qualified Matrix (Corning)-coated wells in 24-well dishes. The hESCs were maintained in hESC medium until 90–100% confluence. On day 0, RPMI medium supplemented with B27 without insulin (RPMI+B27-insulin) and 12 μM CHIR99021 (Stemgent) was added to the culture. After 24 h, the medium was changed to RPMI+B27-insulin. On day 3, RPMI+B27-insulin and 10 μM XAV939 (Sigma) were added to the monolayer cultures, and the cultures were then maintained in this medium until day 5, when the medium was exchanged for RPMI+B27-insulin. Beginning on day 7, the cultures were maintained in RPMI supplemented with B27 complete medium, and the medium was changed every 3 days until day 15. On the final day (day 15), the cells were fixed with 4% paraformaldehyde and stained for cTnT using a previously described immunofluorescence protocol [7]. Twelve fields were photographed at 5x magnification using an Operetta CLS High-Content Analysis System (PerkinElmer). The areas (mm^2^) that showed positive staining for DAPI, eGFP/*NKX2*.*5* and cTnT were calculated using Harmony 4.5 software (PerkinElmer) (S2 Fig). The contraction frequency of the monolayers was manually counted by three individuals who were blinded to the study conditions for each replicate (n = 3).

### Lentiviral vector production and transduction

HEK293FT cells were cultured in Petri dishes containing DMEM supplemented with 10% fetal bovine serum, 1% L-glutamine and 1% penicillin/streptomycin for 24 h. MISSION Lentiviral Mix and a p-LKO1 vector containing a short hairpin RNA (shRNA) targeting *PUM1* (sh*PUM1*; TRCN0000147347, clone 1–15), *PUM2* (sh*PUM2*; TRCN0000061861, clone 2–4) or a scrambled sequence (shSc) (RNAi consortium, Broad Institute, Boston, MA) [31, 32] were added to the cell culture in OptiMEM containing Lipofectamine 2000. After 4 h of incubation, the medium was replaced with supplemented DMEM, as described above. After 48 and 72 h, the medium was collected and centrifuged twice at 141,000 *x g*. The lentiviral pellet was resuspended in 1X PBS and stored at -80°C.

To optimize the transduction efficiency, hESCs were cultured on six-well plates, and different dilutions of the lentiviruses were tested (S1 Fig). A dilution of 10^−3^ was selected for all the experiments. After transduction, the medium was replaced, and hESCs were cultured for 24 h to induce cardiac differentiation.

### RNA extraction and quantitative RT-qPCR

RNA was extracted using an RNeasy Kit (Qiagen), and the cDNA reaction was performed using an Improm-II Kit (Promega) according to the manufacturer’s instructions. Samples were obtained from three replicates of undifferentiated cells and from three independent cardiac differentiations. The cDNA amplification experiments were performed in a final volume of 10 μl containing SYBR Select master mix (Applied Biosystems), 100 ng of the cDNA template, and 5–10 pmol of the primers. The RT-qPCR conditions were performed using the LightCycler system (Roche) in accordance to the manufacturer’s recommendations (Applied Biosystems). The RT-qPCR experiments were performed in triplicate. The Cq results for each gene were normalized based on GAPDH expression, and the relative expression of each gene was calculated. The analyzed genes and the primer sequences are shown in Table 1.

**Table 1:** Primer sets used for RT-qPCR.

Official symbol	NCBI ID	Primer sequence (5´–3´)	Amplicon (bp)
*GAPDH*	NM_002046.3	Forward: 5’-GGCGATGCTGGCGCTGAGTAC-3’	149
		Reverse: 5’-TGGTTCACACCCATGACGA-3’	
*PUM1*	NM_001020658.1	Forward: 5’-AAACCTGAGAAGTTTGAATTG-3’	351
		Reverse: 5’-GCAAGACCAAAAGCAGAGTTG-3’	
*PUM2*	NM_015317	Forward: 5’-AGGATCAGTATGGCAATTATG-3’	389
		Reverse: 5’-ATACTTTTCCAACTTGGCCAG-3’	
*OCT4* (*POU5F1*)	NM_001173531.2	Forward: 5’-ATGCATTCAAACTGAGGTGCCTGC-3’	192
		Reverse: 5’-AACTTCACCTTCCCTCCAACCAGT-3’	
*NANOG*	NM_024865	Forward: 5’-ACCAGAACTGTGTTCTCTTCCACC-3’	200
		Reverse: 5’-CCATTGCTATTCTTCGGCCAGTTG-3’	
		Reverse: 5’-ACAGTGACTGAGCGGCTAAT-3’	

*GAPDH*, glyceraldehyde-3-phosphate dehydrogenase; *PUM1*, Pumilio homolog 1; *PUM2*, Pumilio homolog 2 (Drosophila); *OCT4 (POU5F1)*, Homo sapiens POU class 5 homeobox 1; *NANOG*, Nanog homeobox

### Flow cytometry

During cardiac differentiation, the cells were immunophenotyped by flow cytometry to determine their stages of differentiation. The EBs were dissociated with 0.25% trypsin/EDTA (5 min) and resuspended in PBS/0.5% BSA. On day 3 (D3), the cells were incubated with anti-CD56 (BD) antibody (1:12.5) for 20 min at 4°C. On day 9 (D9), the cells were only dissociated for eGFP detection. On day 15, for cTnT staining, the EBs were incubated with trypsin/0.25% EDTA for 20 min, and this step was followed by inactivation with DMEM supplemented with 50% SFB and DNase I (20–30 U/ml). After dissociation, the cells were fixed with 4% formaldehyde (20 min), permeabilized with 0.5% Triton X-100 (25 min) and incubated with an anti-cTnT primary antibody (Thermo Fisher Scientific) (1:100) for 30 min at room temperature and then with an Alexa Fluor 633 secondary antibody (1:1000) (30 min). The cells were subsequently analyzed using a FACSCanto II (BD) flow cytometer. The data analyses were performed using FlowJo software (v.10).

### Western blot analysis

The cells were washed once with 1X PBS and removed with a cell scraper. The hESCs were then resuspended in SDS sample buffer (160 mM Tris 1 M pH 6.8, 4% SDS, 10% β-mercaptoethanol, 24% glycerol, and 0.02% bromophenol blue). Western blot analyses were performed with goat anti-PUM1 (1:5000, Bethyl Laboratories), rabbit anti-PUM2 (1:2500, Bethyl Laboratories), goat anti-OCT4 (1:500, Abcam) and mouse anti-β-actin (1:1000, Cell Signaling Technology) antibodies. Peroxidase-conjugated anti-goat IgG (1:2500 for anti-PUM1, 1:2000 for anti-OCT4), anti-rabbit IgG (1:2500 for anti-PUM2) and anti-mouse IgG (1:2500 for anti-β-actin) were used as secondary antibodies. HRP Chemiluminescent Substrate Reagent (Thermo Fisher Scientific) was used for chemiluminescence signal generation. The signal was captured with a ChemiExpress L-Pix instrument (Loccus Biotechnology), and the image was generated using L-Pix Image software (v. 2.11.7, Loccus Biotechnology). ImageJ software (https://imagej.nih.gov/ij/) was used for the quantitative analyses, and the gel images were quantified based on the linear signal ranges.

### Immunofluorescence

A previously described immunofluorescence protocol was followed [7]. Briefly, 48 h after the hESCs were transduced with shSc and shPUM1-2, the cells were fixed with 4% paraformaldehyde, rinsed with PBS, and incubated with blocking buffer (PSA/BSA 5%) for 60 min. The cells were subsequently incubated for 60 min at 30°C with primary antibodies for PUM1 (1:300, Bethyl Laboratories Inc.), PUM2 (1:70, Bethyl Laboratories Inc.) or OCT4 (1:100, Abcam), which were diluted in blocking buffer. After three washes with PBS, the cells were incubated with an Alexa Fluor® 488 anti-goat or anti-rabbit secondary antibody for 60 min at 30°C. DAPI staining was performed for 10 min, and the cells were then washed three times with PBS. Images were acquired at 20x magnification using an Operetta CLS High-Content Analysis System (PerkinElmer), and the staining intensity of each individual cell (n = 74,000) was analyzed using Harmony 4.5 software (PerkinElmer) (S2 Fig). The experimental design and control images of the cells incubated with secondary antibody are detailed in S3 Fig (S3A–S3B Fig).

### Analysis of mRNA targets of PUM1 and PUM2 during the early stages of *in vitro* cardiomyogenesis

To analyze the mRNA targets of PUM1 and PUM2 proteins during cardiac differentiation, we defined a set of 1,809 target genes of human Pumilio proteins (PUM1 and PUM2), based on a published study [22] and searched for these in an RNA-seq dataset of mRNAs associated with polysomes obtained during *in vitro* cardiomyogenesis [27]. A differential gene expression (DGE) analysis was performed using the Bioconductor R package edgeR [33,34]. The comparisons were performed among polysome-bound mRNA fractions; specifically, the data from cells at D1 or D4 were compared with data from cells at D0 (undifferentiated hESCs). For these analyses, we retained only those genes with at least one count per million in at least three samples. Based on the initial DGE results, we used a stringent analysis with a p-value threshold of <0.05 and the log 2(fold change) (logFC). Genes with logFC > 1 were considered upregulated, and genes with logFC < −1 were considered downregulated. An enrichment analysis of the identified set of genes was performed using g:Profiler [35] (http://biit.cs.ut.ee/gprofiler/) and the REVIGO [36] (http://revigo.irb.hr/) consortium database, and a complement analysis was performed using STRING Consortium 2019 [37] (https://string-db.org).

### Statistical analysis

The statistical analyses were performed using GraphPad Prism 7 software. The data sets are expressed as the means ± standard deviations. Unpaired Student’s t-test or one-way ANOVA followed by Tukey’s post hoc test were performed when appropriate. Differences with p<0.05 were considered statistically significant.

## Results

### Combined knockdown of *PUM1* and *PUM2* affects the expression of *OCT4* and *NANOG* in hESCs

**Fig 1:**
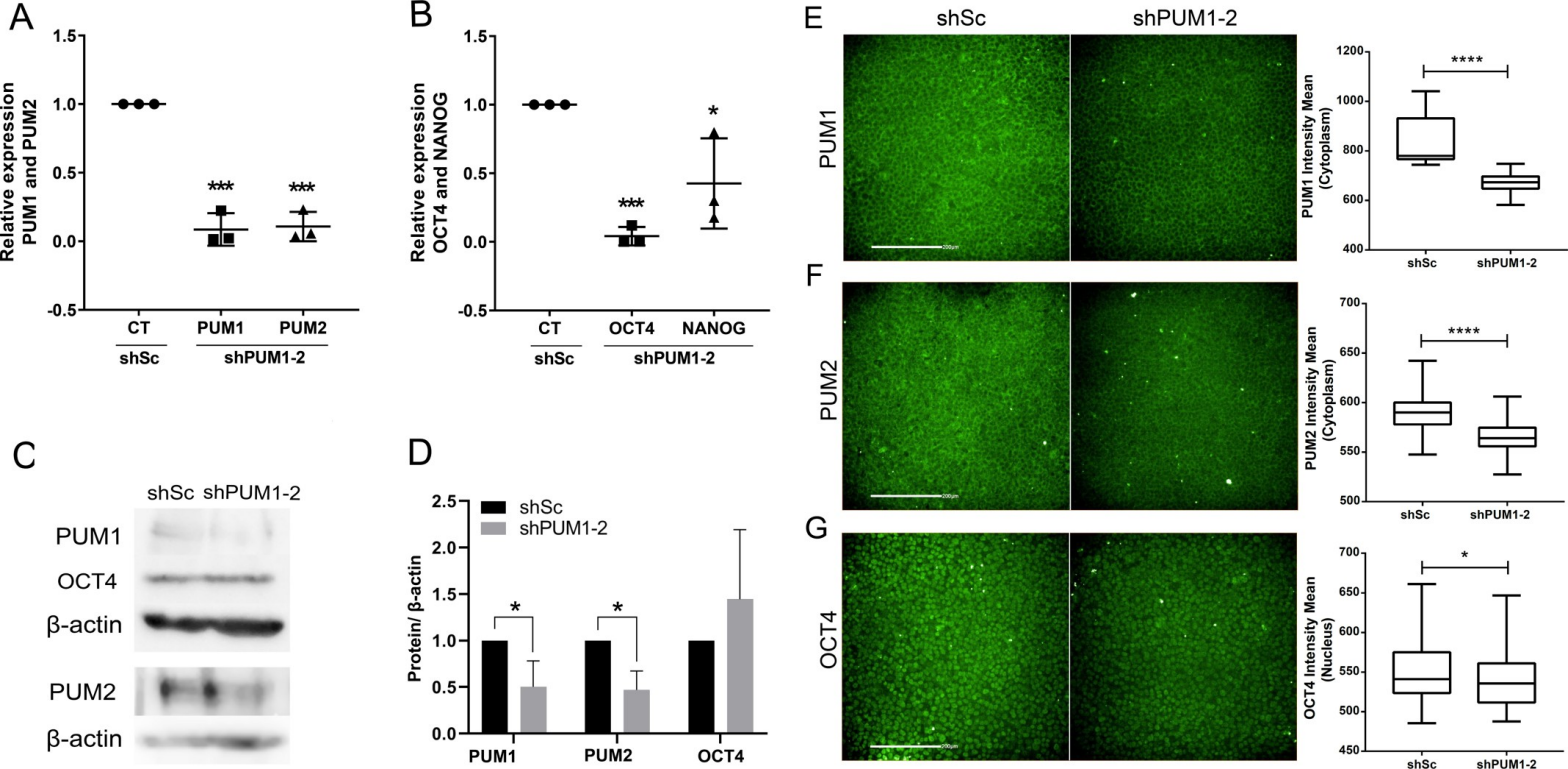
Knockdown of *PUM1* and *PUM2* affects the expression of *OCT4* and *NANOG* mRNA. (A-B) Analysis of the relative expression of *PUM1* and *PUM2* (A) and of *NANOG* and *OCT4* (B) in hESCs 24 h after transduction of lentiviral vectors containing shSc and sh*PUM1-2* (n = 3). (C-D) Western blot analysis of PUM1 (140 kDa), PUM2 (114 kDa) and OCT4 (40 kDa) expression in protein extracts from cells in which *PUM1-2* was silenced. The β-actin (45 kDa) protein was used as a loading control (n = 3). (E-G) The PUM1, PUM2 and OCT4 immunostaining intensity of each individual cell (n = 74,000 cells) was analyzed using Operetta CLS and Harmony 4.5 software (Perkin Elmer); for each sample, 25 images (20X objective) in triplicate were analyzed. Representative immunostaining images and quantified intensity of PUM1 (E), PUM2 (F) and OCT4 (G) 48 h after transduction of lentiviral vectors containing shSc and shPUM1-2 are shown. Scale bars: 200 μm. *p<0.05, **p<0.01, ***p<0.001, ****p<0.0001.

To understand the role of PUM1 and PUM2 in hESC pluripotency, we silenced their expression using short hairpin RNAs. We produced lentiviral particles containing shRNAs that recognize *PUM1* and *PUM2* and a scrambled control; these lentiviruses were based on previously published plasmids [31, 32]. The knockdown of *PUM1* and *PUM2* was confirmed by RT-qPCR, and the results showed that the *PUM1* and *PUM2* mRNA levels were significantly reduced in the double-silenced cells compared with the control cells (Fig 1A). In fact, the results showed that the double-silencing protocol resulted in the efficient (92% and 90%) silencing of the *PUM1* and *PUM2* genes, respectively.

We then analyzed whether the silencing of *PUM1* and *PUM2* altered the mRNA levels of some pluripotency transcription factors, such as *OCT4* and *NANOG*. Interestingly, the mRNA levels of *OCT4* and *NANOG* were significantly reduced in the double-silenced cells compared with the control cells (Fig 1B). We quantified the protein contents in the double-silenced cells by western blot and immunofluorescence of PUM1, PUM2 and OCT4. Both techniques showed that the amount of PUM1 and PUM2 proteins was reduced in the shPUM1-2 transduced cells compared with the level found in the shSc-transduced control cells (Fig 1C and 1D and S3C–S3E Fig). Interestingly, although the western blot analysis indicated that the amount of OCT4 protein remained constant despite the silencing of *PUM1* and *PUM2*, the fluorescence intensity of nuclear OCT4 was reduced in the double-knockdown cells (Fig 1G). However, these results do not allow us to conclude that the reduction in the intensity of OCT4 in the nucleus is directly related to the expression of these proteins. These findings suggest that PUM1 and PUM2 might play some role in pluripotency, and further studies will be needed to understand the relationship between these proteins and OCT4 localization.

### PUM1 and PUM2 expression patterns during *in vitro* cardiac differentiation

**Fig 2:**
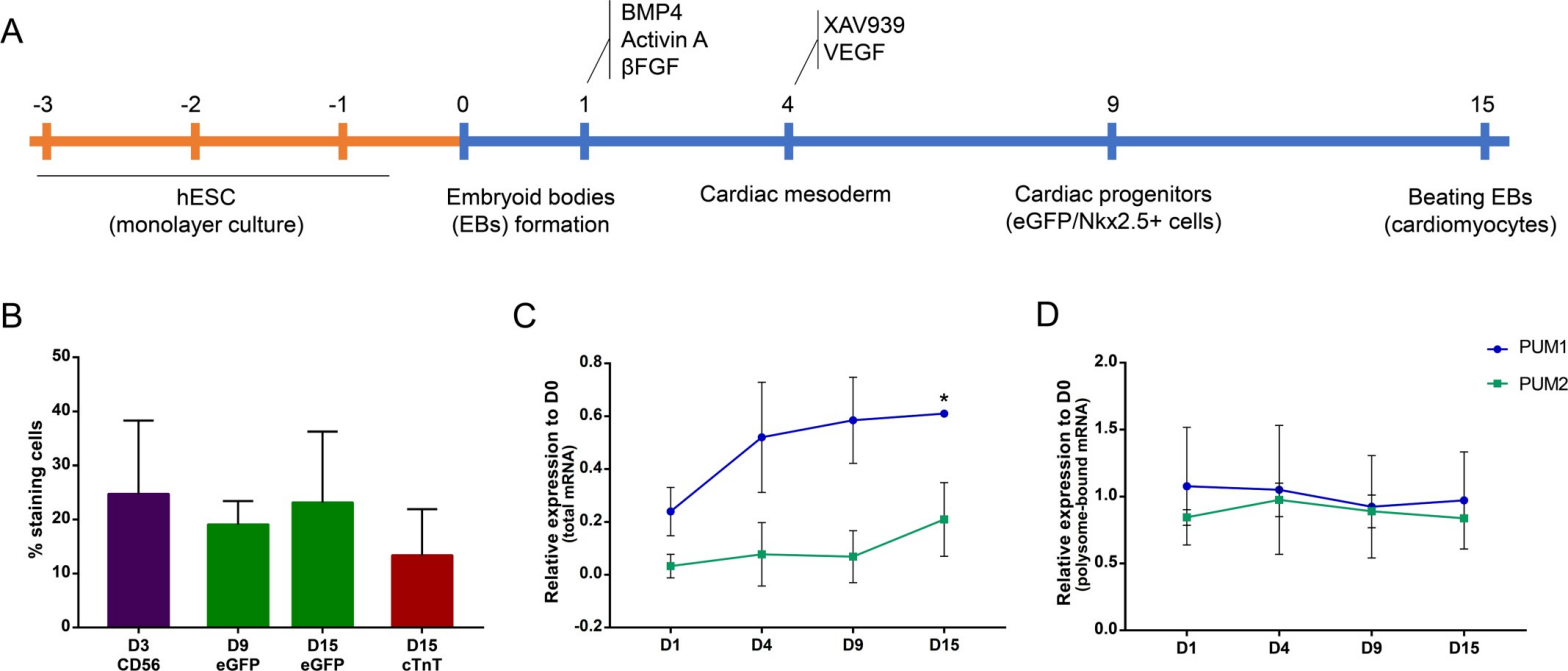
*PUM1* and *PUM2* expression profiles during hESC cardiomyogenesis. (A) Scheme of the EB cardiomyogenic differentiation protocol. (B) Graph indicating the percentage of positive cells for the markers CD56, eGFP/*NKX2*.*5* and cTnT at different time points during *in vitro* cardiac differentiation (n = 3). (C) Relative expression of *PUM1* and *PUM2* at days 1, 4, 9 and 15 of cardiomyogenesis in relation to day 0 (total RNA) (n = 3). (D) Relative expression (RPKM values) of *PUM1* and *PUM2* mRNAs associated with polysomes during *in vitro* cardiomyogenesis in relation to day 0 (data extracted from previously published results [27]). (n = 3) *p<0.05.

After showing that Pumilio proteins might be involved in hESC pluripotency, we investigated their role in a differentiation process: cardiomyogenesis. We evaluated the gene expression levels of *PUM1* and *PUM2* throughout the process of *in vitro* cardiac differentiation. Initially, hESCs were subjected to an *in vitro* cardiac differentiation protocol (Fig 2A), and their progression was verified by flow cytometry. On day 3, approximately 25% of the cell population was CD56+ (mesoderm marker), which indicated that these cells were committed to mesodermal differentiation (Fig 2B). On D9 and D15, the evaluation of eGFP expression, which is under the control of the *NKX2*.*5* promoter (a cardiac progenitor marker), indicated that approximately 20% of the cell population was committed to the cardiac lineage. On day 15, the expression of the cardiomyocyte marker cTnT was assessed, and the results showed that 20% of the cells expressed this marker (Fig 2B).

Total RNA was extracted at five time points during cardiac differentiation (D0, D1, D4, D9, D15) and subjected to RT-qPCR. The results showed that *PUM1* and *PUM2* mRNAs were expressed throughout cardiomyogenesis and that the *PUM1* mRNA level on day 15 was increased compared with that on day 0 (Fig 2C). Using previously reported data from polysome-bound mRNAs during cardiomyogenesis [27], we verified the association of *PUM* mRNAs with polysomes. Based on the fold change in the reads per kilobase million (RPKM) values (comparing each time point in relation to D0), we found that the association of *PUM1* and *PUM2* mRNAs with polysomes also remained constant throughout cardiomyogenesis (Fig 2D). These data suggest that PUM1 and PUM2 might play important roles throughout this cellular process.

### Knockdown of PUM1 and PUM2 in hESCs affects cardiomyogenesis

**Fig 3:**
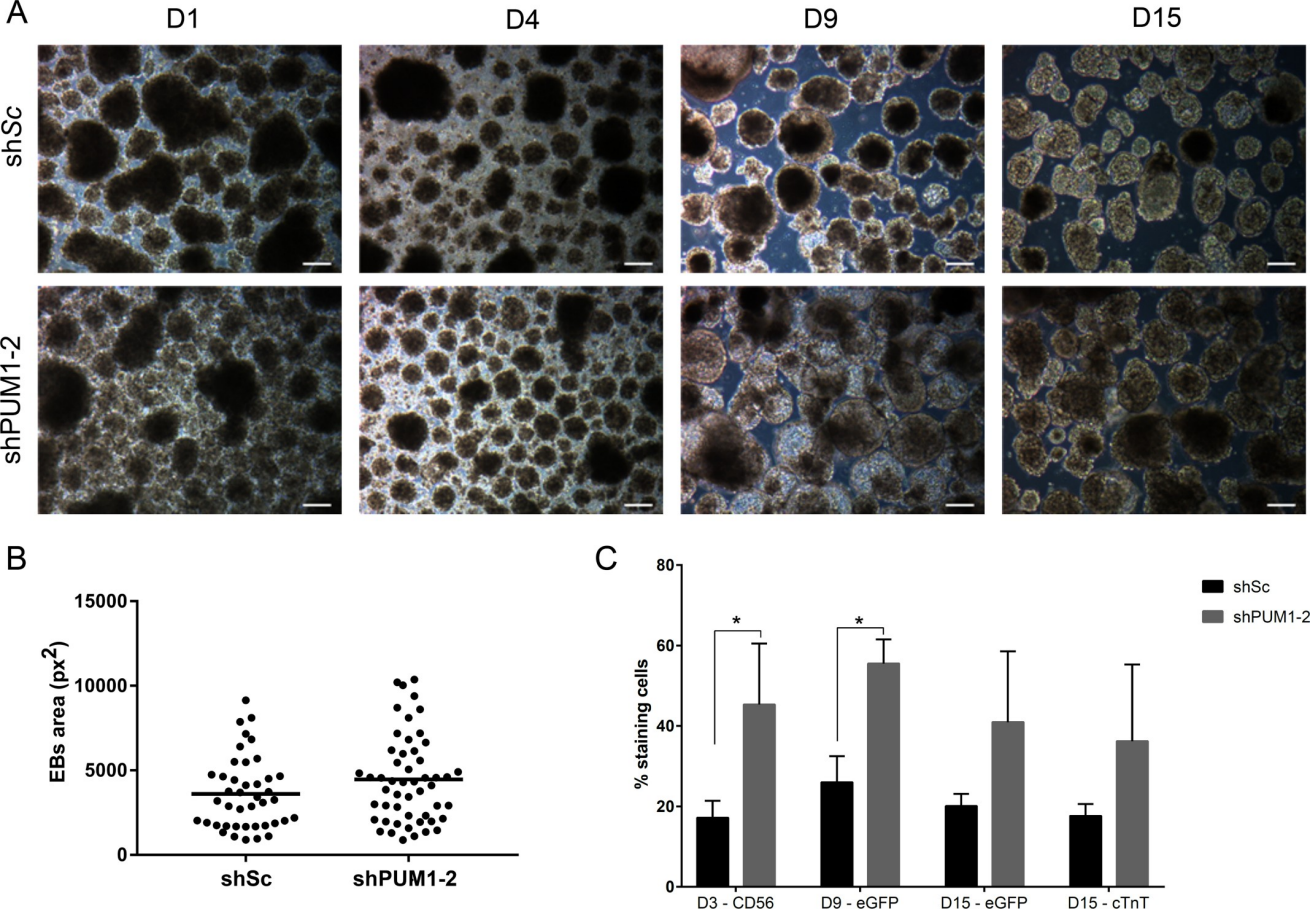
Effect of the knockdown of *PUM1* and *PUM2* during EB cardiac differentiation. (A) Morphology of EBs at days 1 (D1), 4 (D4), 9 (D9) and 15 (D15) of the cardiomyogenic differentiation of hESCs previously transduced with shSc and sh*PUM1-2*. Scale bars: 100 μm. (B) Area of EBs after 9 days of *in vitro* cardiac differentiation (the measurements were performed manually using ImageJ software) (n = 3). (C) Percentage of cells transduced with shSc and sh*PUM1-2* that were positive for CD56, eGFP/*NKX2*.*5* and cTnT during cardiomyogenesis (n = 3). *p<0.05.

Because the silencing of *PUM1* and *PUM2* affected some pluripotency genes (Fig 1) and the expression of *PUM1* and *PUM2* remained constant throughout the process of cardiomyogenic differentiation (Fig 2) (indicating their importance throughout the process), we investigated the influence of silencing Pumilio proteins in the cardiac differentiation process. Forty-eight hour after hESC transduction, EB cardiac differentiation was induced. On day 9, the silenced EBs were morphologically different from the control cells with respect to their size (Fig 3A), although this difference was not statistically significant (Fig 3B). Under all conditions, the EBs contracted spontaneously at D15 (S1 Video).

The analysis of the expression of differentiation markers showed that the numbers of sh*PUM1-2*-transduced cells expressing CD56 and eGFP were increased on D4 and D9 of cardiomyogenesis, respectively, compared with the numbers of control cells (transduced with shSc) (Fig 3C). The analysis of the efficiency of differentiation on D15 showed that the percentage of cTnT^+^ cells did not show a difference between the different treatments (Fig 3C). These results demonstrated that the silencing of *PUM1* and *PUM2* could positively affect the differentiation of mesoderm and cardiac precursors.

**Fig 4:**
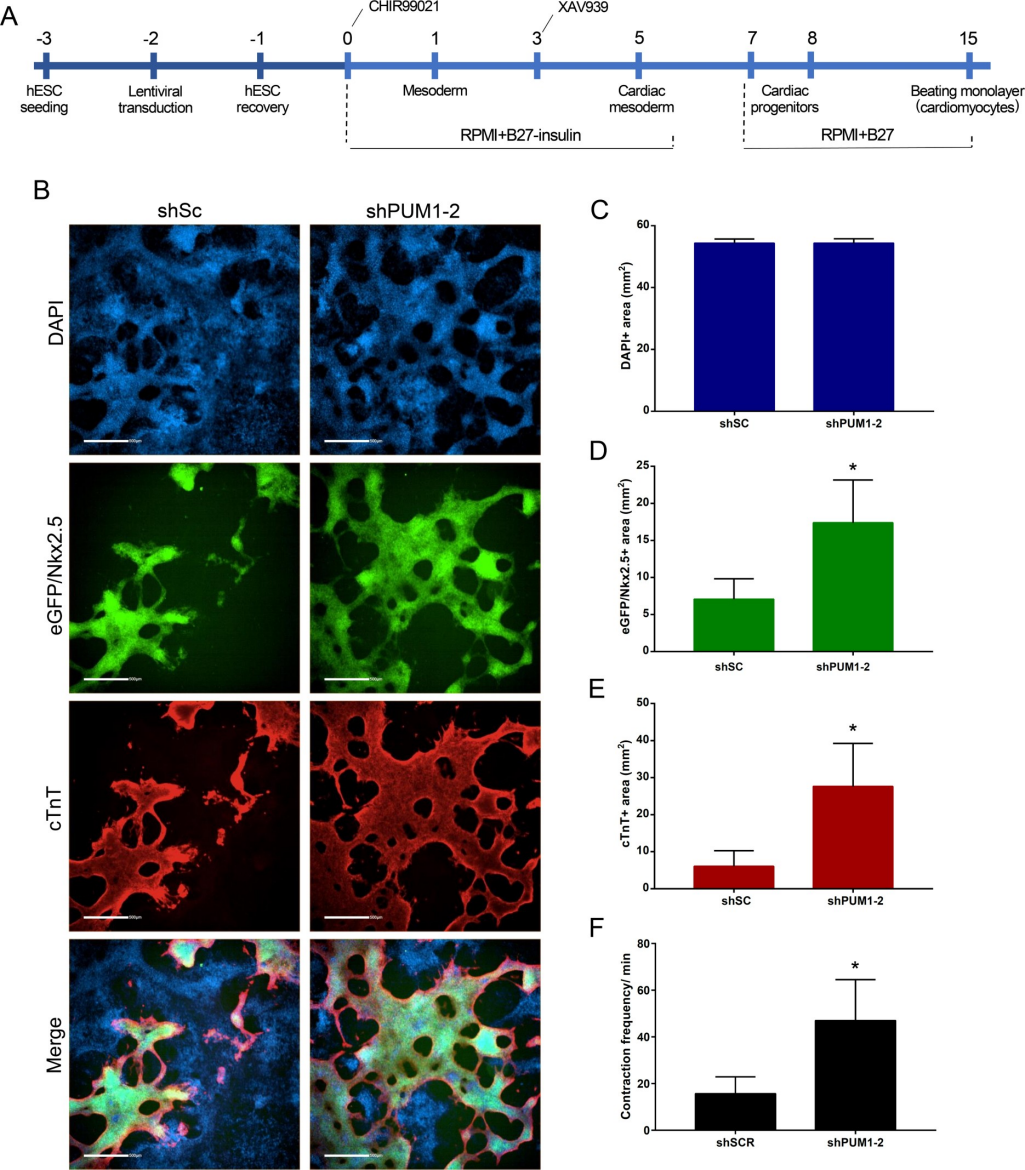
Effect of the knockdown of *PUM1* and *PUM2* during monolayer cardiac differentiation. (A) Scheme of the protocol used for the transduction and monolayer cardiomyogenic differentiation of hESCs. (B) Representative immunofluorescence images of DAPI, eGFP/*NKX2*.*5* and cTnT staining in the population of transduced hESCs after 15 days of monolayer cardiac differentiation. Scale bars: 500 μm. (C-E) The DAPI^+^ (C), eGFP/NKX2.5^+^ (D) and cTnT^+^ (E) stained areas were determined using Operetta CLS and Analysis Software 4.5 (Perkin Elmer) through a sequence analysis of 21 images (5X objective) obtained in triplicate after 15 days of monolayer cardiac differentiation (n = 3). (F) Number of contractions/minute during cardiac monolayer differentiation (n = 3). *p<0.05, **p<0.01.

The dissociation of EBs, particularly at the end of the cardiac differentiation process (D15), is a critical and challenging step. Therefore, the evaluation of eGFP^+^ or cTnT^+^ cells by flow cytometry might underestimate the degree of differentiation and could yield variable results. Thus, we evaluated the effect of the silencing of Pumilio proteins using a monolayer cardiomyogenic differentiation protocol [30] (Fig 4A). Based on this protocol, we transfected hESCs with shSc or sh*PUM1-2* and analyzed their efficiency of cardiac differentiation through an analysis of eGFP/*NKX2*.*5* expression and cTnT immunostaining at day 15. The eGFP^+^ and cTnT^+^ stained areas in the sh*PUM1-2*-treated cell population were significantly higher than those found in the cells transduced with shSc, and a similar number of cells (DAPI^+^ area) were obtained with both treatments (Fig 4B–4E). In addition, the frequency of contractions in the *PUM1-2*-silenced cells after 15 days of differentiation was significantly higher than that in the control cells (shSc) (Fig 4F and S2 Video). Our data suggest that the silencing of *PUM1* and *PUM2* positively affects cardiomyogenesis.

### Identification of mRNA targets of PUM1 and PUM2 during the early stages of cardiac commitment

Our results suggest that PUM1 and PUM2 might play a role during the early stages of cardiomyogenesis because PUM1-2 silencing affects the mesoderm marker (CD56) on day 3 of differentiation (Fig 3C). Thus, we investigated the expression profile of the mRNA targets of PUM1 and PUM2 during the early stages of *in vitro* cardiomyogenesis using previously published RNAseq data of polysome-bound mRNAs obtained during *in vitro* cardiac differentiation [27]. For the DGE analysis, the expression levels of PUM1 and PUM2 mRNA targets on cells at D1 and D4 of differentiation were compared with those at D0 (undifferentiated ESCs) (S1 Table).

**Fig 5:**
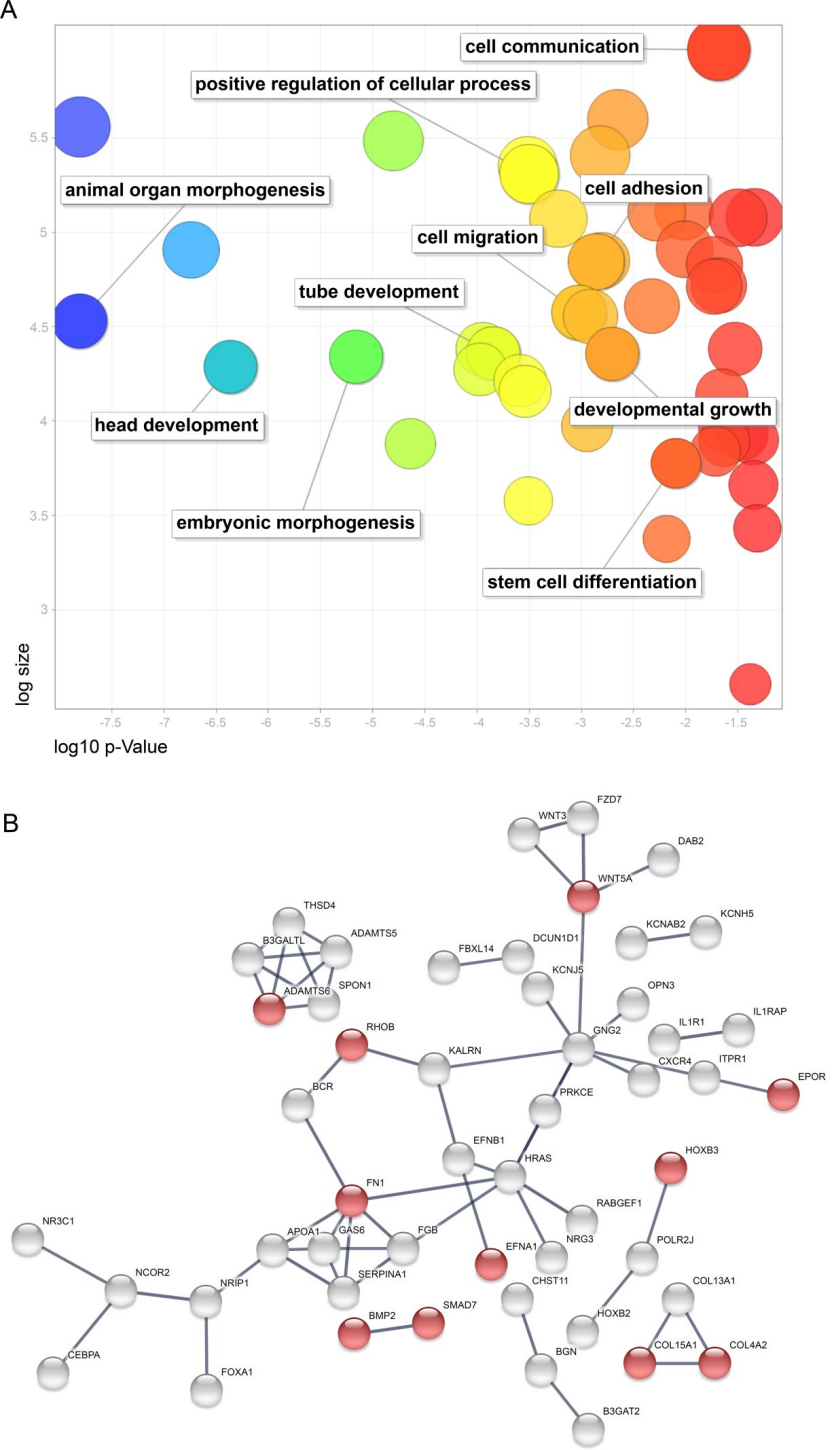
mRNA targets of PUM1 and PUM2 upregulated in the polysome during cardiomyogenesis. (A) Gene ontology analysis of mRNA targets of PUM1 and PUM2 upregulated on day 4 of *in vitro* cardiomyogenesis. The figure shows a REVIGO scatterplot of the representative clusters of GO terms obtained with g:Profiler. In the two-dimensional graph, the log10 p-value of each GO result after REVIGO analysis is plotted on the x-axis, and the terms are scattered based on the log size along the y-axis. The bubble color indicates the provided p-value: bluer colors represent smaller p-values, whereas orange and red colors represent larger p-values. (B) Network analysis of mRNA targets of PUM1 and PUM2 upregulated on day 4 of cardiomyogenesis. Functional enrichments in circulatory system development (FDR 1.99e-05) are represented in red. Interaction Score: Highest confidence (0.900), STRING.

A gene ontology (GO) analysis of each set of PUM1 and PUM2 mRNA targets that were upregulated in polysomes at D1 and D4 was conducted with g:Profiler [35]. The complete lists of cellular components, biological processes, molecular functions and signaling pathways can be found in S2 Table. We observed 69 Pumilio proteins mRNA targets upregulated at D1 in relation to D0, and the highlighted GO terms were related to negative regulation of biological processes, animal organ development and regulation of multicellular organismal processes (S1 and S2 Tables). In contrast, the mRNA targets of PUM1 and PUM2 identified at D4 were associated with more than 120 biological process terms (S2 Table). To aid visualization of these GO terms, we used REVIGO [36]. The highlighted terms are related to involvement in development, stem cell differentiation, embryonic morphogenesis, head development and others (Fig 5A). A more detailed analysis of the animal organ morphogenesis cluster showed terms related to the development of various systems, including the nervous, cardiovascular and circulatory systems (Fig 5A and S2 Table). We also found that these mRNAs were involved in the mesodermal commitment pathway (S2 Table). The network of the interactions between the upregulated mRNA targets of the Pumilio proteins at D4 was then generated, and the genes involved in circulatory system development (Fig 5B, red circles), namely, *EPOR*, *WNT5A*, *EFNA1*, *RHOB*, *HOXB3*, *BMP2*, *SMAD7*, *FN1*, *ADAMTS6*, *COL15A1* and *COL4A2*, are highlighted. Based on the consideration that Pumilio proteins reduce translation and lead to mRNA degradation [15,38,39], some mRNAs related to mesodermal commitment and circulatory system development might have been less repressed; these results indicated that the silencing of *PUM1* and *PUM2* might have improved cardiomyogenesis. Further studies will be needed to understand whether *PUM1* and *PUM2* silencing upregulates mRNAs that participate in cardiomyogenesis.

## Discussion

We investigated the effects of Pumilio proteins on stemness and cardiomyogenesis through the knockdown of both human paralogs. Twenty-four hours after the silencing of *PUM1* and *PUM2*, hESCs showed a reduction in the mRNA levels of two genes associated with the pluripotency phenotype, *OCT4* and *NANOG*. This finding indicated that PUM proteins might be involved in the maintenance of hESC pluripotency. Our results corroborate those reported by Uyhazi et al. (2020), who showed that the double knockout of *Pum1* and *Pum2* in mESCs leads to decreases in the Oct4, Sox2 and Nanog protein levels [40]. Other studies also strongly suggest that PUF proteins might mediate a widespread and ancient mechanism for repressing the differentiation and maintaining the self-renewal of stem cells [10,15,4–44].

The evaluation of the OCT4 protein levels in the *Pum1*-and-*Pum2*-silenced cells through western blot and immunofluorescence analyses showed distinct results: one of the analysis showed no changes in the protein content, whereas the other indicated a reduction in the staining intensity. This finding was also observed by Lin et al. (2018), who analyzed mouse E6.5 *Pum1-2* double-knockout embryos and found decreases in both the overall Oct4 signal intensity by immunohistochemistry staining and the total amount of Oct4 mRNA, but amount of total Oct4 protein was constant by western blot assays [45]. Human PUM1 and PUM2 bind with high affinity and specificity to the Pumilio response element (PRE) with the consensus binding site 5′-UGUANAUA (where N is A, C, G or U) [46–48]. Bohn et al. (2018) identified diverse target RNAs that are functionally regulated by human Pumilio proteins in HEK293 cells, but OCT4 and NANOG are not included among these targets and do not contain the PRE recognition motif [49]. Thus, the silencing effect of Pumilio proteins on pluripotency genes might occur indirectly. Furthermore, because PUM1 and PUM2 have not been described as OCT4-binding partners [50–52], it is possible that the silencing of Pumilio proteins acts indirectly by regulating some mRNAs that encode proteins responsible for the translocation of OCT4 out of the nucleus. After analyzing whether any mRNA targets of PUM1 and PUM2 encode proteins that form part of the OCT4 interactome [50], we found that the following mRNAs associate with one or both Pumilio proteins: *MLLT10*, *RFC4*, *TBP*, *PPP1R10*, *DBT*, *HK2*, *MLLT4*, *SMARCA2*, *WDR5* and *RIF1*. Further studies will be needed to understand whether these elements are related to OCT4 localization and pluripotency.

By investigating the gene expression profile of *PUM1* and *PUM2* throughout the cardiac differentiation process, we realized that the mRNA for both genes was constantly associated with polysomes, which indicated that maintenance of the *PUM1* and *PUM2* mRNA levels is important for cell function and cardiac differentiation processes. The importance of PUM1 and PUM2 in different types of stem cells and lineage differentiation was previously demonstrated. The protein levels of PUM2 exhibit no changes during the differentiation of human adipose-derived stem cells into adipocytes [53]. The mouse *Pum1* and *Pum2* genes show differential expression in fetal and adult hematopoietic stem cells and progenitors [12]. The repression of Pumilio protein expression by Rbfox1 promotes germ cell differentiation in Drosophila [54].

Considering the possible role of Pumilio proteins in cardiac differentiation, we examined the effect of their silencing on hESCs subjected to *in vitro* cardiomyogenesis. After the EB protocol, *PUM1-2*-silenced hESCs showed increased percentages of CD56^+^ cells (D3) and eGFP^+^ cells (D9), even though the differences in the percentages of eGPF^+^ and cTnT^+^ cells at D15 was not statistically significant. It is important to note that the percentage of positive cells during the EB cardiac differentiation protocol depends on the dissociation of EBs that contain organized cell-cell junctions, which makes the quantification of single cells by flow cytometry difficult. Other studies have also reported difficulty in the dissociation of pluripotent stem cell (hPSC)-derived cardiomyocytes [55].

Moreover, using the monolayer protocol, we observed that *PUM1-2*-silenced hESCs exhibited a larger cTnT-stained area compared with the control cells. These changes indicated that the reduction of *PUM1* and *PUM2* expression, even over a short period, can increase the number of cardiomyocytes generated through *in vitro* cardiac differentiation protocols. Therefore, our results indicate that the knockdown of *PUM1-2* improves cardiac differentiation. Lin et al. (2018) showed that PUM1 and PUM2 double-knockout ESCs exhibit spontaneous differentiation; although pluripotency factors such as OCT4, NANOG and REX1 were not affected, the ectoderm marker Nestin was significantly reduced, and the endoderm markers GATA6 and LAMA1 were significantly increased [45]. Uyhazi et al. (2020) showed that the knockout of *Pum2* in mESCs during spontaneous differentiation increased the mesoderm and resulted in an earlier appearance of beating EBs compared with wild-type EBs, although these effects were not observed in EBs in which both *Pum1* and *Pum2* were knocked out [41]. This difference compared with our data might be due to fact that the previous study analyzed spontaneous differentiation, whereas we analyzed induced differentiation toward a specific cell lineage.

A previous study found that EBs in which both *Pum1* and *Pum2* were knocked down increased in size after 12 days [41]. Although our visual inspection indicated that the EBs in our study also appeared larger, the quantification of the EB area did not reveal a significant difference. Interestingly, *in vivo* analyses can yield different findings. Mice in which *Pum1* and/or *Pum2* are knocked out present smaller body and organ sizes than control mice, and this decrease in dose-dependent [41, 56]. Gennarino et al. (2018) also showed that different PUM1 levels in *PUM1*-haploinsufficient patients can cause neurodisorders, such as ataxia, seizures and smaller cerebellum, and these effects are dose-dependent. These researchers also analyzed the expression of some PUM1 targets and found that AAMP, a protein involved in angiogenesis, appears to be elevated in patients with lower PUM1 expression [57]. However, the effects on the heart appear to be contradictory. Lin et al. (2019) showed that the size and weight of the hearts of *Pum1*-knockout mice do not differ from those of wild-type mice [56]. Another study showed that the hearts from *Pum1*-knockout mice are smaller than those from wild-type mice [41]. These size-related effects might be related to the fact that Pumilio proteins regulate mRNAs that encode proteins involved in the regulation of the cell cycle [56, 57]. Further studies are needed to determine the effect of Pumilio on heart development and growth and the effects of different Pumilio expression levels can affect these processes.

Based on our results, the effects of *PUM1-2* silencing might occur at the beginning of the differentiation process, which results in a larger number of cardiomyocytes at the end of the process. Thus, in an attempt to understand the mechanism that occurs in *PUM1-2*-silenced cells, we investigated the targets of PUM1 and PUM2 at the early stages of the cardiomyogenic differentiation process and focused on the mesoderm stage.

The analysis was performed using data from mRNAs associated with polysomes obtained during cardiomyogenesis. The enrichment of mRNAs in polysomal fractions indicated the translation of these genes [27]. At D4 of the cardiac differentiation process, we found a group of genes that were enriched in the polysomal fraction and contained Pumilio recognition elements. Therefore, we postulate that the expression of Pumilio-targeted mRNAs would be more prone to translation after the silencing of these proteins, which would promote cardiomyogenesis.

Among the Pumilio-targeted mRNAs, we found some that exhibit a strong correlation with the cardiac development process. For example, BMP2 was previously shown to play a crucial role in early cardiomyogenesis by inducing *NKX2*.*5* expression in the precardiac mesoderm [58, 59]. The noncanonical WNT signaling protein WNT5A, which is also a target of Pumilio, regulates cardiovascular development and functional cardiomyocyte differentiation [60]. In mice, cell signaling orchestrated by fibronectin (FN1) plays indispensable roles in cardiovascular development [61]. The expression of erythropoietin receptor (EpoR) is not restricted to the erythroid lineage. Mice lacking erythropoietin receptor expression suffer from ventricular hypoplasia and exhibit a reduced number of proliferating cardiac myocytes [62]. Therefore, it is possible that the role of the Pumilio in the control of cell differentiation is to regulate the availability of genes associated with cell fate in the translation machinery, which results in control of the process in a time-dependent manner.

Despite this regulatory role, the mechanism underlying the effects observed in *PUM1-2*-silenced hESCs needs to be investigated. The influence on cardiac differentiation might be related to decreases in pluripotency markers or the modification of marker localization. Additionally, our study focused on cardiac commitment, but the silencing of Pumilio proteins could potentiate differentiation toward other lineages, such as endoderm and ectoderm. The mRNA targets identified at the initial stages of cardiomyogenesis also indicated that Pumilio can act early during the process of differentiation, but further studies will be needed to understand whether PUM1 and PUM2 silencing upregulates these mRNAs during cardiac lineage commitment.

Our results suggest that *PUM1-2* affects the expression of pluripotency genes as well as the efficiency of the cardiac differentiation process, which corroborates the findings detailed in the literature. Our study contributes to the understanding of the roles of Pumilio proteins in the maintenance of pluripotency and the differentiation processes of hESCs.

## Supporting information

S1 Fig Selection by puromycin and lentiviral titration. A) Morphological analysis of cells after treatment with puromycin for 7 days. The positive cell viability control (CTRL^+^) constitutes cells with no drug administered (0 ng/ml). Scale bars: 100 μm. B) Graph depicting the cell viability detected based on neutral red after the addition of puromycin. The positive cell viability control (CTRL^+^) refers to cells with no drug administered (0 ng/ml), and the negative cell viability control (CTRL^-^) constitutes cell-free wells without any drug administration. C-E) Titration of the lentiviral vectors. C) Number of resistant colonies after transduction with lentiviral vectors containing sh*PUM1*. D) Number of resistant colonies after transduction with lentiviral vectors containing sh*PUM2*. E) Number of resistant colonies after transduction with lentiviral vectors containing shSc.
(DOCX)

S2 Fig Operetta HCS analysis layout. A) The PUM1, PUM2 and OCT4 immunostaining intensity was determined using Operetta CLS and Harmony Software 4.5 (Perkin Elmer) through a sequence analysis of 25 images (20X objective) in triplicate. B) The eGFP/*NKX2*.*5-* and cTnT-immunostained areas were determined using Operetta CLS and Harmony Software 4.5 (Perkin Elmer) through a sequence analysis of 21 images (5X objective) in triplicate.
(DOCX)

S3 Fig Analysis of PUM1, PUM2, and OCT4 proteins. A) Experimental design for the immunofluorescence assay. Cells without lentiviral transduction were used as the control. B) Control images of cells incubated with secondary antibody. Scale bars: 400 μm. (C-E) Western blot analysis of PUM1 (C), PUM2 (D) and OCT3/4 (E) in shSc- and sh*PUM1-2*-transduced cells; three replicates of each condition were included. The bands that were used to create Fig 1C are outlined.
(DOCX)

S1 Table Differentially expressed mRNA targets of PUM1 and PUM2. “FC4vs0_pvalue<0.05”: Differentially expressed mRNA targets of PUM1 and PUM2 in polysomes at day 4 vs day 0 of the cardiomyogenesis of hESCs. “FC1vs0_pvalue<0.05”: Differentially expressed mRNA targets of PUM1 and PUM2 in polysomes at day 1 vs day 0 of the cardiomyogenesis of hESCs.
(XLSX)

S2 Table Gene ontology analysis of mRNA targets of PUM1 and PUM2 upregulated at days 1 and 4 of cardiomyogenesis. The GO terms were obtained with g:Profiler.
(XLSX)

S1 Video Beating embryoid bodies transduced with shSc (left) and sh*PUM1-2* (right) after 15 days of cardiac differentiation. (MP4)

S2 Video Beating monolayer of hESCs transduced with shSc (left) and sh*PUM1-2* (right) after 15 days of cardiac differentiation. (MP4)

S1 File (DOCX)
