# Supplementary material for: Effects of PUMILIO1 and PUMILIO2 knockdown on cardiomyogenic differentiation of human embryonic stem cells culture
Source: PLoS One. 2020 May 21;15(5):e0222373. doi: 10.1371/journal.pone.0222373 (PMC7241771; doi:10.1371/journal.pone.0222373)
Supplement: S1 Fig — A) Morphological analysis of cells after treatment with puromycin for 7 days. The positive cell viability control (CTRL+) constitutes cells with no drug administered (0 ng/ml). Scale bars: 100 μm. B) Graph depicting the cell viability detected based on neutral red after the addition of puromycin. The positive cell viability control (CTRL+) refers to cells with no drug administered (0 ng/ml), and the negative cell viability control (CTRL-) constitutes cell-free wells without any drug administration. C-E) Titration of the lentiviral vectors. C) Number of resistant colonies after transduction with lentiviral vectors containing shPUM1. D) Number of resistant colonies after transduction with lentiviral vectors containing shPUM2. E) Number of resistant colonies after transduction with lentiviral vectors containing shSc. (DOCX) [file pone.0222373.s001.docx]

**FUNCTION OF PUMILIO GENES IN HUMAN EMBRYONIC STEM CELLS AND THEIR ROLE IN STEMNESS AND CARDIOMYOGENESIS**

Silva, I.L.Z. et al.


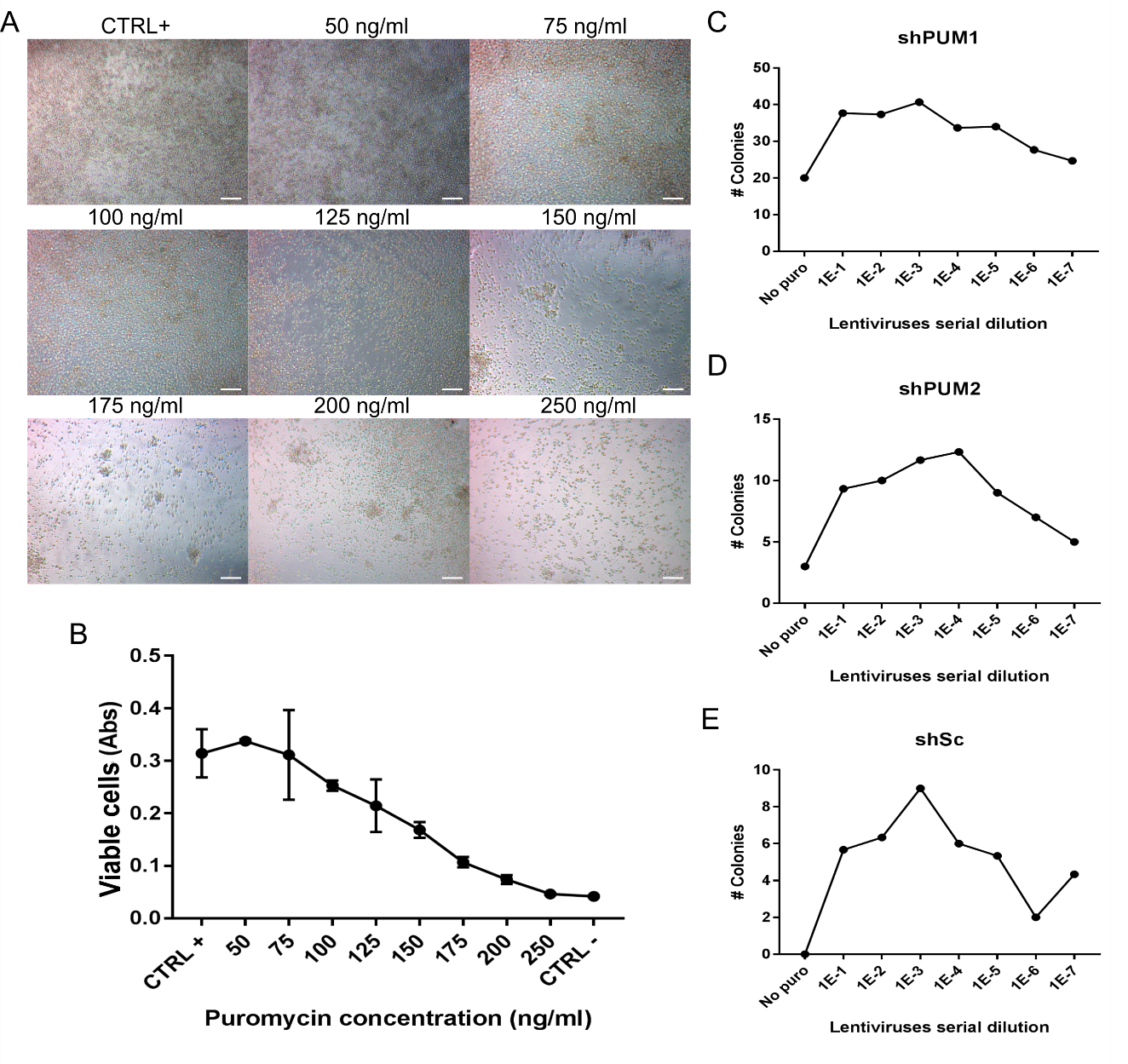


**S2 Fig. Selection by puromycin and lentiviral titration.**

A) Morphological analysis of cells after 7 days of puromycin treatment. Positive cell viability control (CTRL+) shows cells with no drug administered (0 ng/ml). Scale bars: 100 μm. B) Graph depicting cell viability with neutral red after the addition of puromycin. Positive cell viability control (CTRL+) shows cells with no drug administered (0 ng/ml). The cell viability negative control (CTRL-) shows a cell-free well and no drug administration. C-E) Titration of the lentiviral vectors. C) Number of resistant colonies after transduction with lentiviral vectors containing shPUM1. D) Number of resistant colonies after transduction with lentiviral vectors containing shPUM2. E) Number of resistant colonies after transduction with lentiviral vectors containing shSc.
