# Supplementary material for: Effects of PUMILIO1 and PUMILIO2 knockdown on cardiomyogenic differentiation of human embryonic stem cells culture
Source: PLoS One. 2020 May 21;15(5):e0222373. doi: 10.1371/journal.pone.0222373 (PMC7241771; doi:10.1371/journal.pone.0222373)
Supplement: S2 Fig — A) The PUM1, PUM2 and OCT4 immunostaining intensity was determined using Operetta CLS and Harmony Software 4.5 (Perkin Elmer) through a sequence analysis of 25 images (20X objective) in triplicate. B) The eGFP/NKX2.5- and cTnT-immunostained areas were determined using Operetta CLS and Harmony Software 4.5 (Perkin Elmer) through a sequence analysis of 21 images (5X objective) in triplicate. (DOCX) [file pone.0222373.s002.docx]

**FUNCTION OF PUMILIO GENES IN HUMAN EMBRYONIC STEM CELLS AND THEIR ROLE IN STEMNESS AND CARDIOMYOGENESIS**

Silva, I.L.Z. et al.


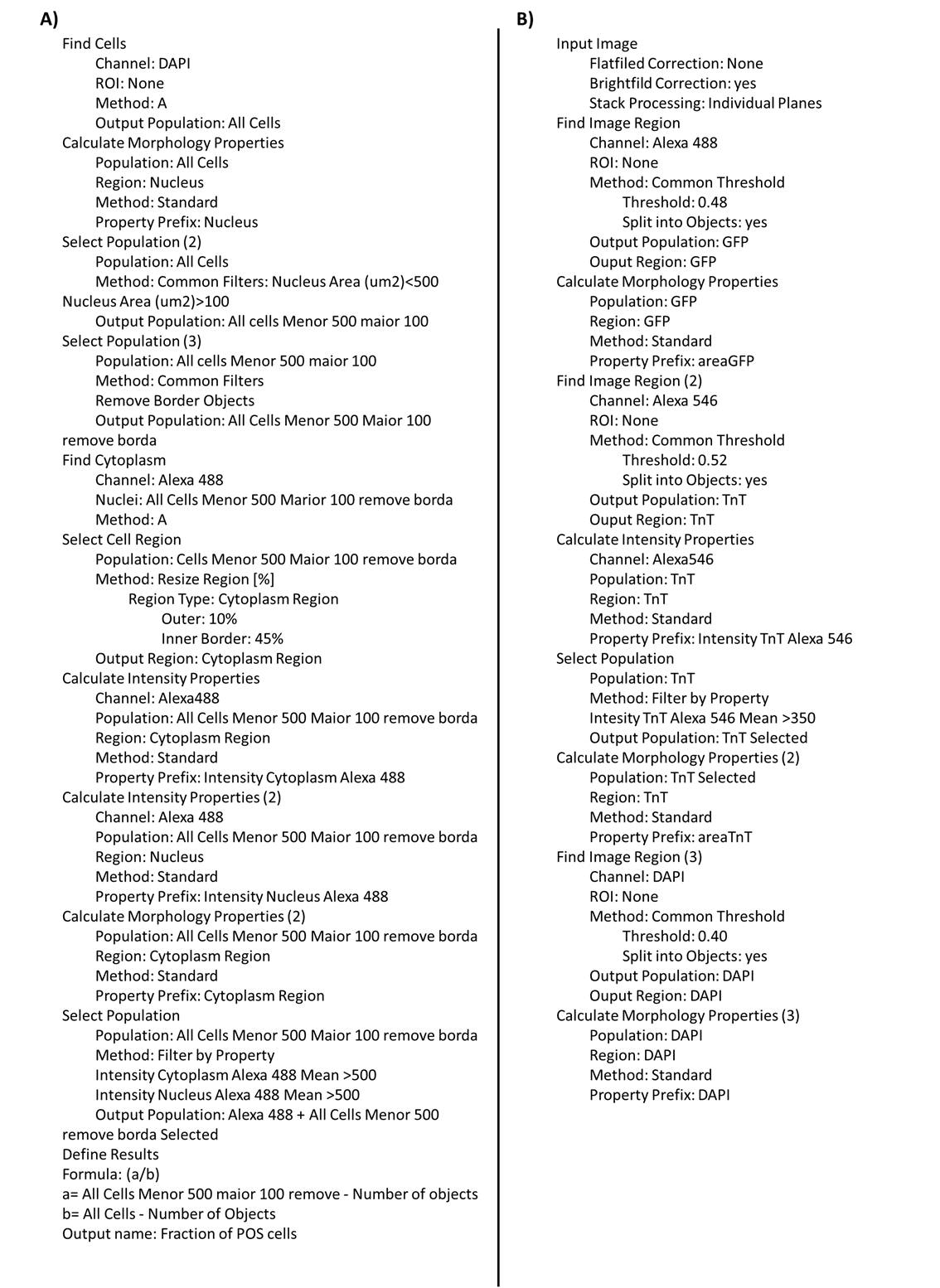


**S2 Fig. Operetta HCS analysis layout.** A) Determination of PUM1, PUM2 and OCT4 immunostaining intensity was performed using Operetta CLS and Harmony Software 4.5 (Perkin Elmer) by analysis sequence in 25 images (20X objective) in triplicate. B) Determination of eGFP/*NKX2.5* and cTnT immunostaining area were performed using Operetta CLS and Harmony Software 4.5 (Perkin Elmer) by sequence analysis of 21 images (5X objective) in triplicate.
