# Supplementary material for: Effects of PUMILIO1 and PUMILIO2 knockdown on cardiomyogenic differentiation of human embryonic stem cells culture
Source: PLoS One. 2020 May 21;15(5):e0222373. doi: 10.1371/journal.pone.0222373 (PMC7241771; doi:10.1371/journal.pone.0222373)
Supplement: S3 Fig — A) Experimental design for the immunofluorescence assay. Cells without lentiviral transduction were used as the control. B) Control images of cells incubated with secondary antibody. Scale bars: 400 μm. (C-E) Western blot analysis of PUM1 (C), PUM2 (D) and OCT3/4 (E) in shSc- and shPUM1-2-transduced cells; three replicates of each condition were included. The bands that were used to create Fig 1C are outlined. (DOCX) [file pone.0222373.s003.docx]

**FUNCTION OF PUMILIO GENES IN HUMAN EMBRYONIC STEM CELLS AND THEIR EFFECT IN STEMNESS AND CARDIOMYOGENESIS**

Silva, I.L.Z. et al.


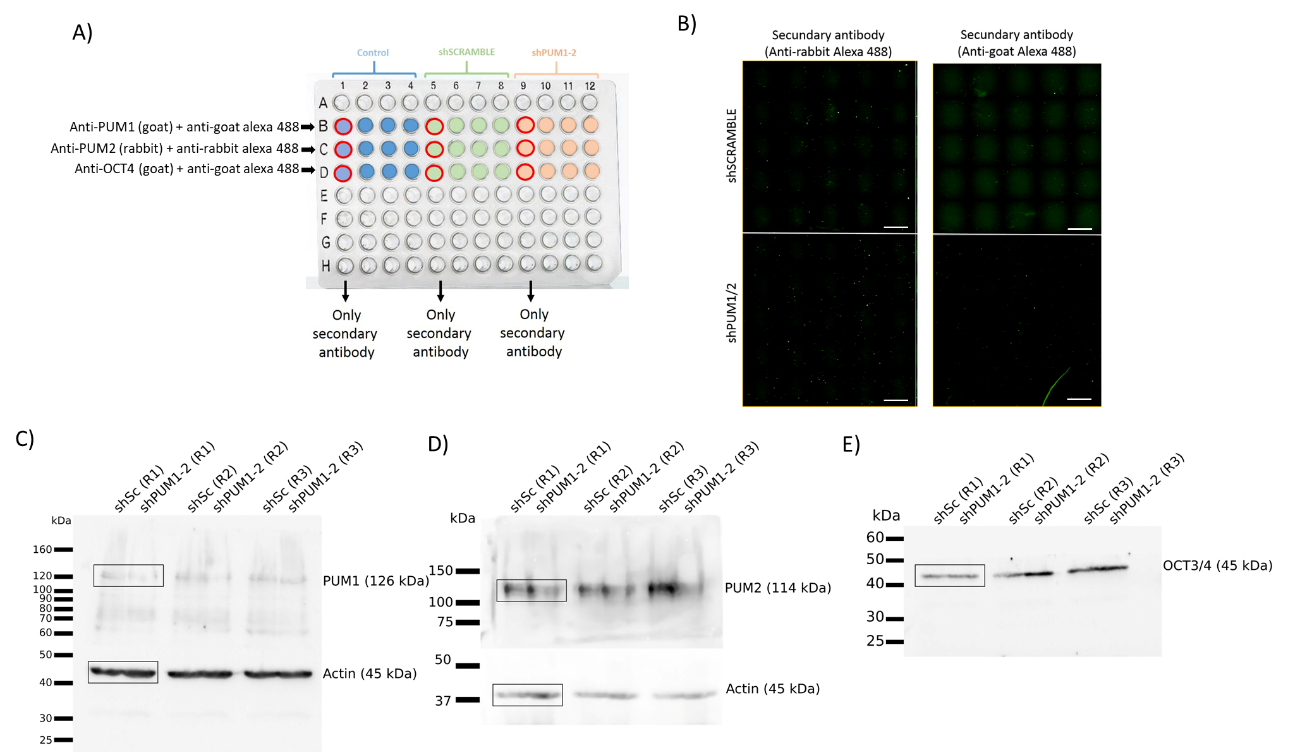


**S3 Fig. Protein analysis for PUM1, PUM2, and OCT4.** A) Experimental design for immunofluorescence assay. Control was cells without lentiviral transduction. B) Control images of cells incubated with secondary antibody. Scale bars: 400 μm. (C-E) Western blot for PUM1 (C), PUM2 (D) and OCT3/4 (E) in shSc and shPUM1-2, with three replicates each. The bands that were used to create the Figure 1C are outlined.
