# Supplementary material for: Effects of PUMILIO1 and PUMILIO2 knockdown on cardiomyogenic differentiation of human embryonic stem cells culture
Source: PLoS One. 2020 May 21;15(5):e0222373. doi: 10.1371/journal.pone.0222373 (PMC7241771; doi:10.1371/journal.pone.0222373)
Supplement: S1 File — (DOCX) [file pone.0222373.s008.docx]

**FUNCTION OF PUMILIO GENES IN HUMAN EMBRYONIC STEM CELLS AND THEIR EFFECT IN STEMNESS AND CARDIOMYOGENESIS**

Silva, I.L.Z. et al.


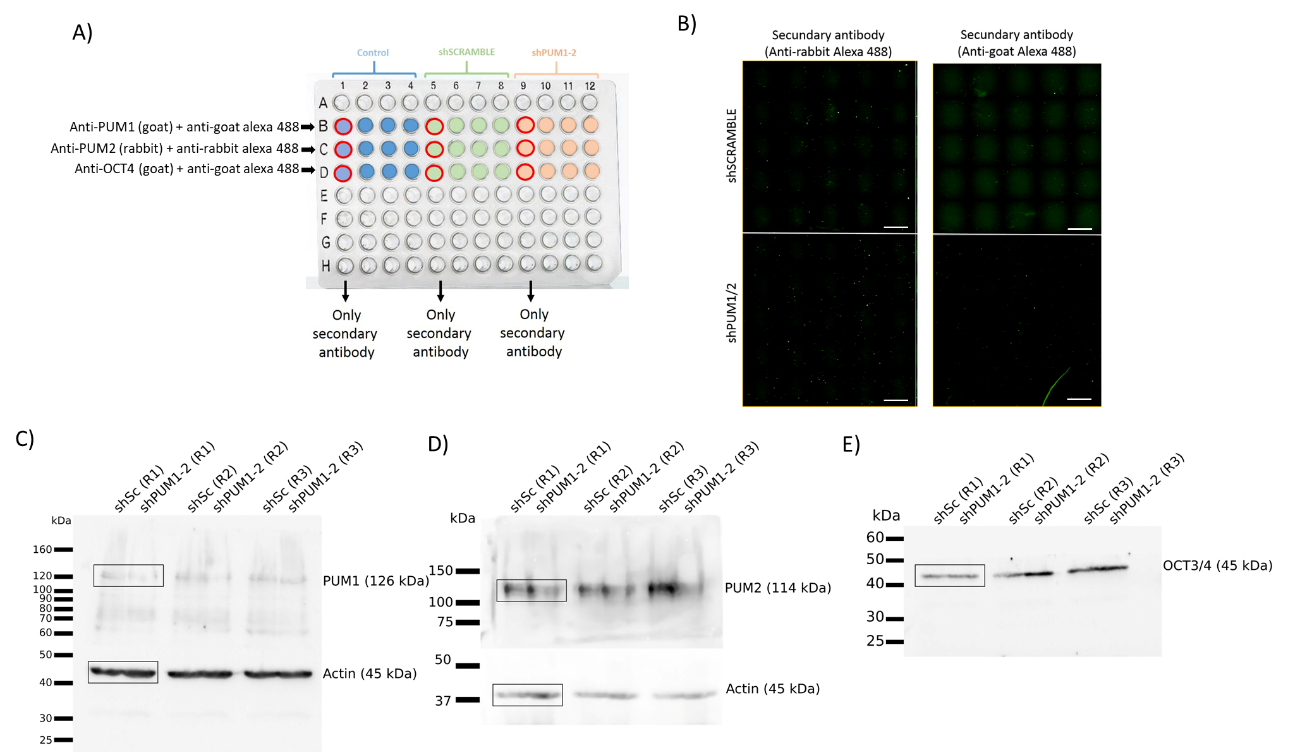


**S3 Fig. Protein analysis for PUM1, PUM2, and OCT4.** (C-E) Western blot for PUM1 (C), PUM2 (D) and OCT3/4 (E) in shSc and shPUM1-2, with three replicates each. The bands that were used to create the Figure 1C are outlined.
